# A framework for collaborative wolverine connectivity conservation

**DOI:** 10.1016/j.isci.2021.102840

**Published:** 2021-07-10

**Authors:** Kathleen A. Carroll, Robert M. Inman, Andrew J. Hansen, Rick L. Lawrence, Kevin Barnett

**Affiliations:** 1 Montana State University, Ecology Department, Montana State University, PO Box 173460, Bozeman, MT 59717, USA; 2 University of Wisconsin-Madison, Department of Forest and Wildlife Ecology, 1630 Linden Dr, Madison, WI 53706, USA; 3 Montana Fish, Wildlife and Parks, 1420 E 6th Avenue, Helena, MT 59620, USA; 4 Montana State University, Land Resources and Environmental Sciences Department, 334 Leon Johnson Hall, P.O. Box 173120, Bozeman, MT 59717, USA; 5 The Wilderness Society, 503 W Mendenhall St, Bozeman, MT 59715, USA

**Keywords:** Earth sciences, Ecology, Environmental science, Nature conservation

## Abstract

Maintaining connectivity between high-elevation public lands is important for wolverines and other species of conservation concern. This work represents the first effort to prioritize wolverine connectivity under future climate conditions using a systematic conservation planning framework. We optimized 10, 15, 20, and 50% of habitat features for wolverines using integer linear programming. We identified 369 privately owned areas in the 10% solution, 572 in the 15% solution, 822 in the 20% solution, and 3,996 in the 50% solution where voluntary landowner easements would improve the long-term landscape functionality for wolverine connectivity. The median estimated easements ranged from $8,762 to $12,220 across the four solutions (total costs $14,874,371 to $196,346,714). Overall, this effort demonstrates the utility of optimization problems for conserving connectivity, provides a proactive tool to engage potential collaborators, identifies easements that will likely protect various subalpine species, and offers a framework for the conservation of additional species.

## Highlights

• Voluntary landowner easements would improve long-term wolverine connectivity
• Total costs ranged from $14,874,371 to $196,346,714
• Total number of private areas ranged from 369 to 3,996 across four solutions
• Connectivity is essential for wolverine persistence, recovery, and reestablishment

## Graphical abstract

**Figure:**
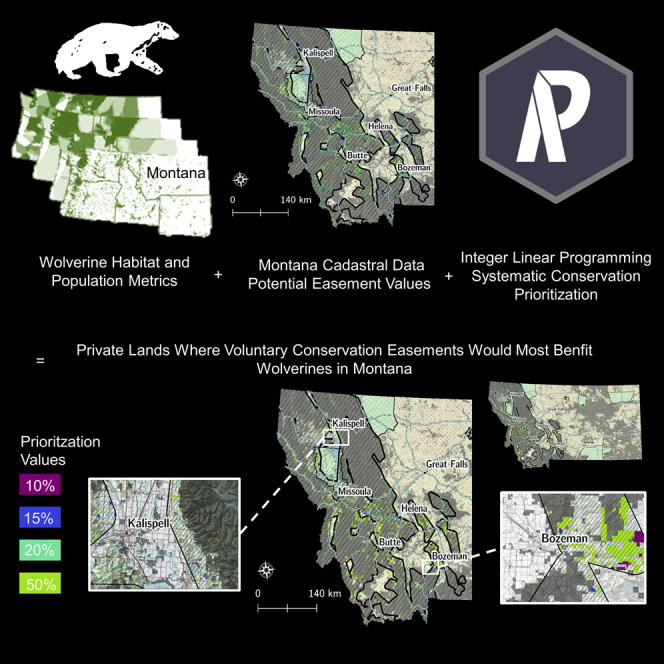

## Introduction

In the Rocky Mountain West (RMW), protected conservation areas and long-term wildlife conservation have historically focused on high-elevation systems with little economic or agricultural value (Scott et al., 2001; Joppa and Pfaff, 2009). This focus has resulted in conservation areas being unbalanced, with well-represented high-elevation ecosystems but less well-represented low-elevation ecosystems (Scott et al., 2001; Dietz and Czech, 2005; Aycrigg et al., 2013). While high-elevation conservation areas are highly valuable for species conservation, they are isolated and require species to move down through private lands to maintain natural ecological processes. Many RMW species (e.g., elk, mule deer, pronghorn, wolverines, bighorn sheep) migrate or disperse across land tenures with varying degrees of protection throughout their lifetime (Sawyer et al., 2009; Allen et al., 2016; Tack et al., 2019; Carroll et al., 2020; Middleton et al., 2020).

Due to the vast area and various land tenures in the RMW, including complex relationships between humans and wildlife in multiuse systems, building a balanced network of conservation lands is a complicated issue (Elbroch et al., 2020). Over the last 30 years, the RMW has experienced an economic transition resulting in further rural and exurban development (Ahmed and Jackson-Smith, 2019; Runge et al., 2019). Urbanization and development threaten both wildlife and rural communities whose economies depend on open space (Hamilton et al., 2016; Braunstein et al., 2020). For many broad-ranging species and rural communities, continuing development directly affects their resilience and persistence (Johnson et al., 2017). Thus, successfully conserving rural private land in the RMW can mutually benefit wildlife species and rural communities. Private land conservation efforts depend on identifying the most valuable areas to conserve and building partnerships with local landowners to develop the tools and support necessary to conserve rural landscapes.

For many species, such as wolverines (*Gulo gulo*), species persistence and continued recovery to historical range hinge on successful dispersers or migrants crossing low-elevation private lands (Cegelski et al., 2006) and therefore private land conservation. Across the western US, a few hundred wolverines reside primarily in high-elevation public lands. Because territorial behavior dictates how individuals partition space in areas with limited resources (Haenel et al., 2003), only a few wolverines may be found within any mountain range. As such, the young often disperse out of their natal mountain range, across privately owned land in valley bottoms, and into a neighboring mountain range where they establish their territory (Inman et al., 2012). Successful dispersal is critical for the species to continue occupying the available habitats and maintaining genetic diversity in the conterminous US (Kyle and Strobeck, 2001; Cegelski et al., 2006).

Proactively identifying, prioritizing, and incentivizing conservation in areas that provide the most benefit for successful dispersal by wolverines would be a significant step toward conserving this species (Inman et al., 2013). These conservation efforts would also benefit other RMW carnivores and rural RMW communities. Thus, in the RMW, positive landowner-agency collaborations are necessary to mitigate wolverine habitat fragmentation, isolation, and loss (Knight and Cowling, 2007). Conservation easements (i.e., voluntary legal agreements that limit land use for conservation) are an essential tool to facilitate natural species dispersal and migration, as easements maintain open space on private land. The number of voluntary conservation easements in the RMW is rapidly increasing, and voluntary easements are critical to protecting many wide-ranging species across the West (Smith et al., 2016; Quintas-Soriano et al., 2020).

Protecting private lands using voluntary conservation easements is increasingly crucial in landscape connectivity and conservation (Smith et al., 2016; Belote et al., 2017; Bargelt et al., 2020). However, there is far more spatial variability in land value than in the associated biodiversity benefits (Naidoo et al., 2006). Despite this, many conservation priority schemes are economically inefficient (Game et al., 2013; Brown et al., 2015). By ignoring the monetary cost of conservation actions, conservation biologists sacrifice efficiency with their often limited conservation resources (Naidoo et al., 2006). The cost-effectiveness of conservation actions is contingent on considering costs together with conservation benefits and landowners' willingness to collaborate in conservation efforts. If costs to incentivize landowner cooperation are incorporated into conservation analyses, such as return on investment analyses, conservation planning benefits per dollar are greater than returns from conservation plans that ignore these considerations (Naidoo et al., 2006; Boyd et al., 2015).

Balancing the three primary factors defining an area's conservation priority, namely, biodiversity value, threat level, and private land easement cost, is incredibly challenging (Sacre et al., 2019). Using a prioritization approach to incorporate all three factors allows researchers to systematically and defensibly identify solutions that adhere to minimum conservation targets (Margules and Pressey, 2000). Furthermore, when cadastral data are available for questions focused on minimizing costs, identifying areas eligible for conservation action fits well in an optimization problem framework (Beyer et al., 2016). Mathematical optimization provides a clear and more defensible framework for designating areas or lands requiring conservation action based on ecological importance and financial constraints. Optimization models and software allow researchers to maximize efficiency and identify areas that, if protected, allow practitioners and policymakers to meet conservation goals under limited budgets (Beyer et al., 2016). Optimizing investments made in private land conservation is, thus, crucial given the challenges in establishing conservation easements under financial constraints.

Here, we employed a prioritization approach to identify which areas would be most beneficial to facilitate wolverine connectivity in western Montana (USA). Our goal was to prioritize private lands to maintain and expand the wolverine metapopulation connectivity while accounting for the economic constraints of agency and land trust. We developed alternative conservation scenarios to evaluate how the optimal set of private lands varies with gradients of conservation targets. This analysis can guide conservation practitioners seeking to facilitate continued wolverine persistence, recovery, and reestablishment, building upon previous efforts to identify areas necessary for wolverine connectivity (Dilkina et al., 2017). We used future climate projections to provide additional, complementary information to previous efforts and ensure our findings will be valuable over the long term for conservation efforts in conjunction with private landowners.

## Results

**Figure 1:**
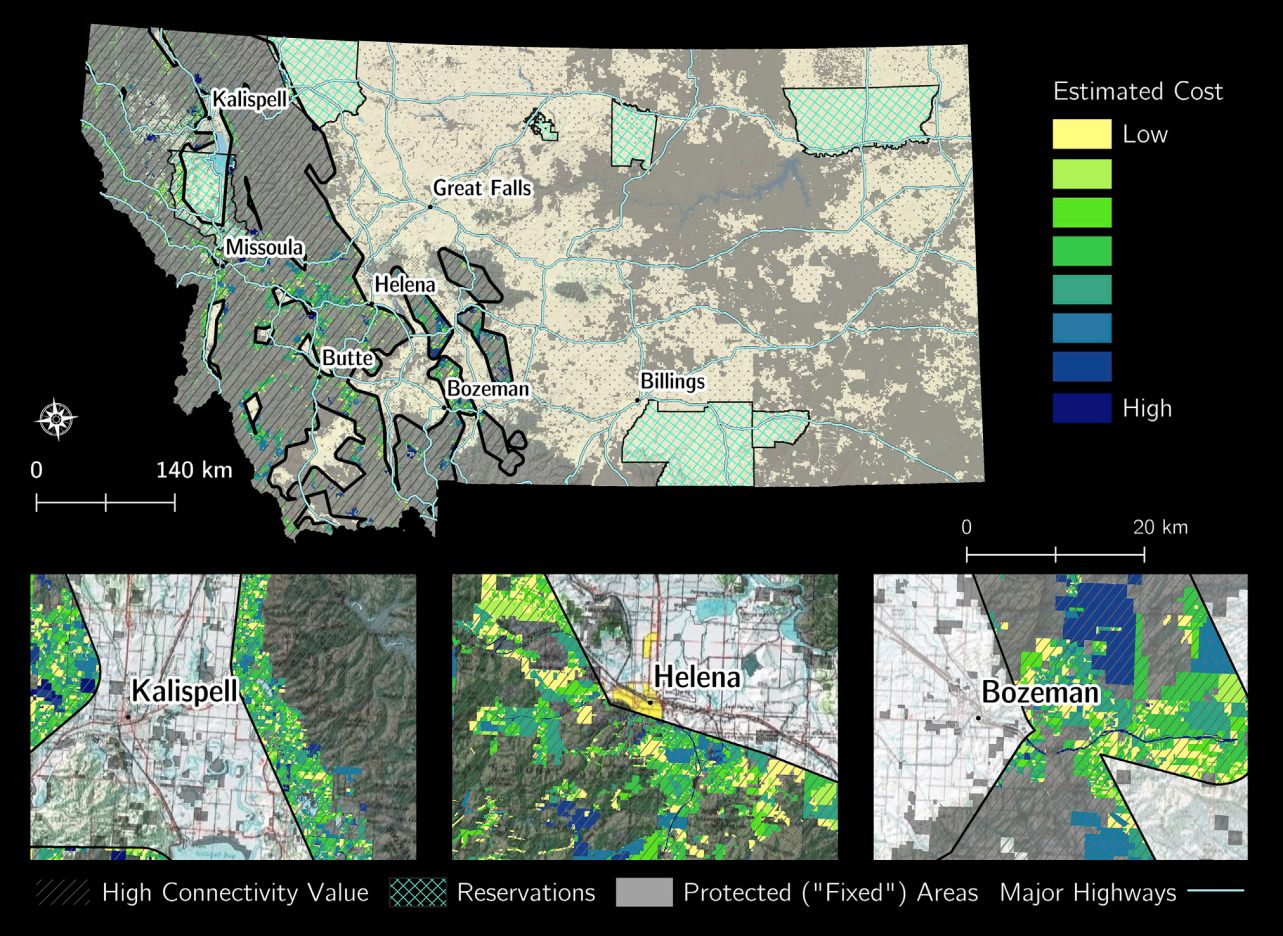
Montana cadastral data potential easement values (www.cadastral.mt.gov).

**Figure 2:**
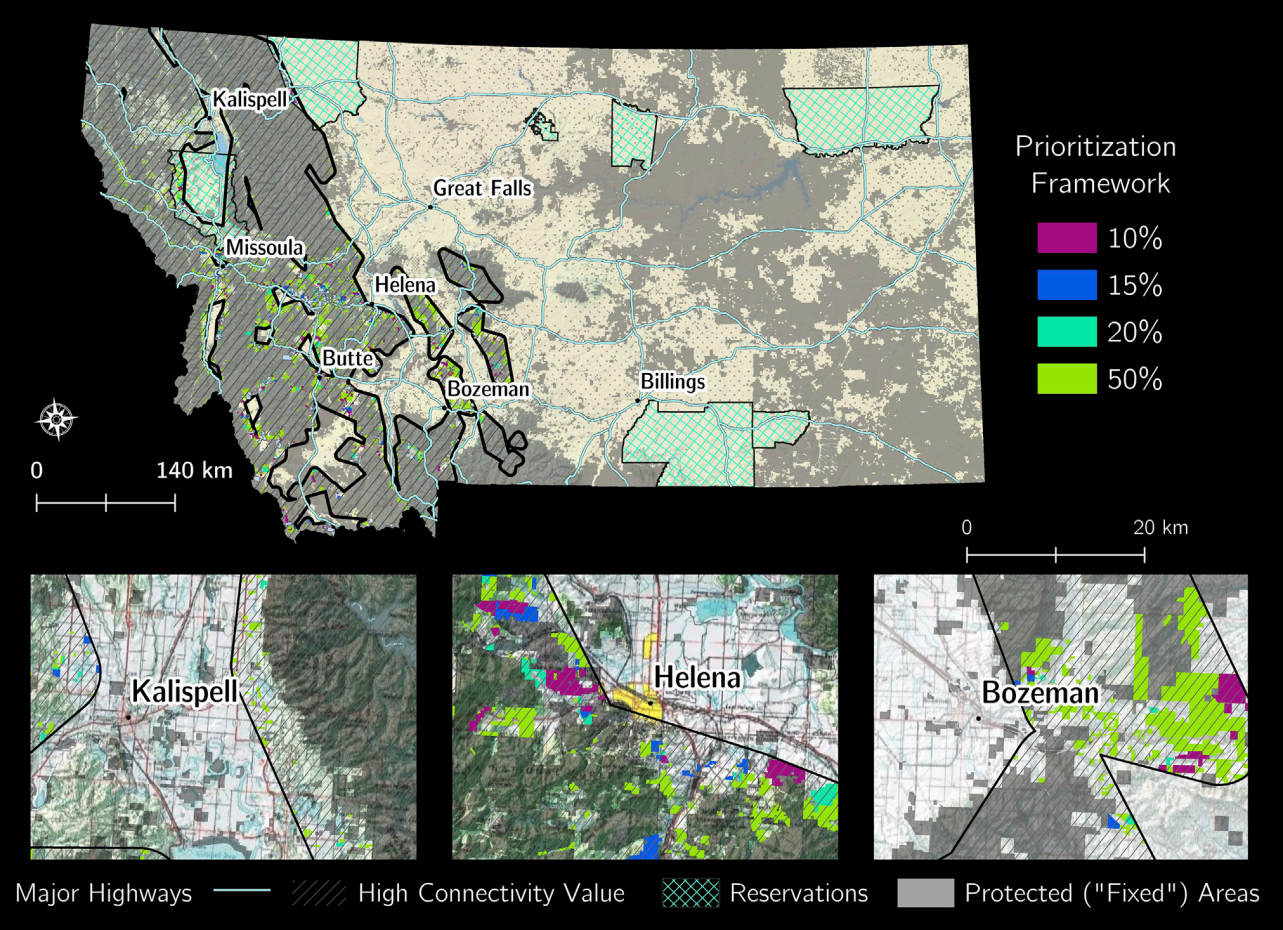
Optimization output for the top 10%, 15%, 20%, and 50% frameworks within high connectivity value areas See Table S2 for land cover types associated with each solution.

Areas of potential easements and high conservation priority for future wolverine connectivity were distributed throughout 26 counties in the study area (Figures 1 and 2). Most counties examined had at least one private land area identified in each solution (Table 1). Many of the privately owned areas identified were adjacent to cities in western Montana. We also identified important areas on tribal land, adjacent to major highways and bordering “fixed” parcels (Figure 2).

**Table 1:** The number of identified privately owned areas that would be valuable for voluntary conservation easement by county and solution

County	10% count	15% count	20% count	50% count
Beaverhead	45	64	77	189
Broadwater	14	20	25	86
Cascade	2	4	5	29
Deer Lodge	19	23	29	141
Flathead	0	4	10	122
Gallatin	22	27	54	314
Glacier	16	23	31	62
Granite	17	32	48	200
Jefferson	12	23	32	170
Judith Basin	0	0	1	2
Lake	7	15	18	72
Lewis and Clark	17	32	45	240
Lincoln	1	3	5	46
Madison	42	56	90	453
Meagher	13	15	18	88
Mineral	2	4	4	67
Missoula	26	54	76	368
Park	16	17	28	186
Pondera	0	0	2	3
Powell	27	41	58	280
Ravalli	35	58	89	494
Sanders	15	25	38	208
Silver Bow	20	30	36	157
Sweet Grass	1	1	2	9
Teton	0	0	0	8
Wheatland	0	1	1	2
Sum	369	572	822	3996

In our solutions, the 10% model identified 369 areas, the 15% model identified 572 areas, the 20% model identified 822 areas, and the 50% model identified 3,996 areas across 26 counties where voluntary easements could benefit wolverine connectivity conservation (Table 1). The estimated easement cost for incentivized easements ranged from $5,073 to $969,934 across all solutions with medians of $12,076 for the 10% solution, $12,145 for the 15%, $12,220 for the 20%, and $8,762 for the 50%, and means ranged from $40,310 to $49,136 (Table 2). The large difference between the mean and medians resulted from a positive skew in the values of individual areas. We estimated that the total cost of acquiring all identified areas as incentivized easements ranged from $14,874,371 to $196,346,714 (Table 2).

**Table 2:** Estimated incentivized easement costs for each solution

Priority level	Estimated easement (USD)				
	Min	Max	Mean	Median	Total solution cost
10%	5,425.00	836,737.00	40,310.00	12,076.00	14,874,371.00
15%	5,335.00	836,737.00	46,380.00	12,145.00	26,529,421.00
20%	5,260.00	836,737.00	49,425.00	12,220.00	40,627,075.00
50%	5,073.00	969,934.00	49,136.00	8,762.00	196,346,714.00

We also examined which areas were selected by the optimization criteria on a county-by-county basis to aid managers that may only be working in a limited number of counties. We found that Beaverhead, Madison, and Ravalli County had the most extensive areas of important connective areas located on private land across the four solutions (Table 3). In the study area, counties included 58–99% public lands (median = 79%). Counties with higher percentages of public lands generally had fewer identified potential easements. Across the 26 counties containing areas of high conservation priority for future wolverine connectivity, 4%–44% of private lands were both within the study area and our solutions for the 10% and 50% solutions, respectively (Table 3).

**Table 3:** The area in each county important for wolverine connectivity (hectares) and the proportion held in private land for each prioritization solution

County	10% (ha)	Per area private	15% (ha)	Per area private	20% (ha)	Per area private	50% (ha)	Per area private
Beaverhead	7692.06	0.04	8019.45	0.05	50,940.45	0.30	96,823.17	0.56
Broadwater	815.51	0.02	1259.66	0.03	5196.8	0.11	20,597.53	0.45
Cascade	294.8	0.04	440.35	0.07	761.99	0.11	3385.81	0.51
Deer Lodge	6138.69	0.11	6138.69	0.11	14,369.33	0.27	27,064.19	0.50
Flathead	0	0.00	116.16	0.00	363.04	0.00	4520.36	0.02
Gallatin	1906.72	0.02	1906.72	0.02	6588.15	0.07	28,541.53	0.30
Glacier	1155.47	0.03	1155.47	0.03	4902.87	0.11	6473.87	0.15
Granite	3758.73	0.05	4796.54	0.06	19,380.26	0.24	58,456.95	0.72
Jefferson	883.62	0.01	1142.21	0.01	9884.86	0.12	27,495.51	0.34
Judith Basin	0	0.00	0	0.00	64.71	0.20	113.86	0.34
Lake	149.64	0.00	149.64	0.00	542.54	0.01	3862.84	0.06
Lewis and Clark	2406.18	0.03	2406.18	0.03	9475.18	0.13	26,618.79	0.36
Lincoln	63.25	0.00	133.64	0.00	260.44	0.00	2264.84	0.01
Madison	3283.45	0.03	5670.46	0.05	22,974.85	0.21	46,682.06	0.43
Meagher	4110.89	0.04	4110.89	0.04	8641.64	0.09	41,889.09	0.42
Mineral	92.63	0.00	155.9	0.00	248.53	0.01	4445.48	0.13
Missoula	1811.49	0.01	1811.49	0.01	8150.65	0.04	28,452.8	0.14
Park	2145.82	0.04	2145.82	0.04	6921.79	0.11	22,674.91	0.37
Pondera	0	0.00	0	0.00	47.53	0.02	80.33	0.03
Powell	8213.9	0.05	8892.53	0.06	36,103.57	0.24	80,735.1	0.53
Ravalli	850.13	0.01	3382.37	0.04	10,503.05	0.12	28,005.3	0.32
Sanders	801.52	0.00	801.52	0.00	5711.36	0.03	21,140.3	0.10
Silver Bow	599.03	0.01	5233.31	0.11	9845.84	0.21	23,687.06	0.50
Sweet Grass	60.92	0.00	60.92	0.00	84.51	0.01	5167.52	0.31
Teton	0	0.00	0	0.00	0	0.00	1419.01	0.50
Wheatland	0	0.00	316.01	0.18	316.01	0.18	1353.45	0.75
Sum	47,234.47		60,245.93		232279.95		611951.63	

In the 10% solution, per private land area, Deer Lodge County had the highest number of identified privately owned land where seeking private landowner collaborations would be valuable (Table 3). While we only identified 19 areas in Deer Lodge County, this land represents 11% of the privately owned land in the county (Tables 1 and 3). After Deer Lodge County, Granite and Powell Counties both had approximately 5% of private lands identified as areas where landowner collaborations would benefit wolverines. When moving to the 50% solution, which has less stringent requirements for the inclusion of private lands for the variables examined, Wheatland, Granite, Beaverhead, Powell, Cascade, Deer Lodge, Silver Bow, and Teton Counties all had 50% or more of private lands identified as areas where landowner collaborations would benefit wolverines.

## Discussion

This work is one of the first applications of optimization software with species-level data to identify future high-priority conservation areas. Ensuring connectivity between high-elevation habitat patches is essential to facilitate wolverine and other RMW species persistence, recovery, and reestablishment. Notably, many high-value connectivity areas for wolverines already have some degree of protection in Montana (Figure 2). Thus, for wolverines and many other species, maintaining connectivity to facilitate natural species dispersal is likely more critical for species persistence than ensuring individuals are present in any given mountain range or isolated area (Perry and Lee, 2019).

Our four output maps with the 10, 15, 20, and 50% of high-value areas enabled us to generate a hierarchy of important private lands across Montana for wolverine conservation (Figure 2). Because each subsequent map has less stringent requirements for private land inclusion, these outputs balance ecological importance with flexible planning. If efforts to engage with landowners begin with the 10% solution, which has the lowest overall cost of incentivizing landowner participation and the smallest land area, the most important connective areas that mitigate human threats (e.g., development) and maintain ecological processes (e.g., dispersal) under future climate conditions would be conserved. Alternatively, the 15%, 20%, and 50% solutions provide additional areas that are still relatively important for mitigating human threats and maintaining ecological processes, but less effectively than the areas identified in the 10% solution (i.e., rank of solution effectiveness is 10% > 15% > 20% > 50%).

We expected a larger number of high-priority areas in all of our optimal solutions (Table 1, Figure 2). However, the broad extent of public or “fixed” lands appeared to limit the number of high-priority areas. This finding, paired with our previous work demonstrating that wolverines are less sensitive to low habitat quality during dispersal than previously thought, indicated that there may be more flexibility in implementing wolverine connectivity conservation than previously recognized (Carroll et al., 2020). This finding represents a considerable improvement for the wolverine conservation planning outlook. Together with other conservation initiatives, the use of these solutions can help managers and private landowners jointly build a more resilient and well-connected wolverine population over the coming decades that benefits numerous other RMW species. In the future, it would be interesting to include additional species of conservation concern in this analysis to benefit a broad array of connectivity for wide-ranging species.

### Implementation

Our results provide a novel and valuable toolset for wolverine conservation planning. Effective collaborations between landowners and agencies are critical to balance nuanced conservation planning problems and protect the large number of at-risk species on private lands while also considering the budgetary constraints faced by the organizations and agencies involved (Baier, 2020). These maps are only a small portion of a much greater conservation effort and should be seen as a tool to aid conservation practitioners. Thus, we recommend that practitioners work synergistically with private land partners to select conservation areas based on our framework and their localized knowledge of the region. In the Midwest, for example, conservation actions were more likely to succeed if there was already planning at the local agency level and efforts built on past conservation activities (Carter et al., 2015). Thus, with this toolset, a local-level agency plan, and knowledge of previous easement successes, on-the-ground conservation for wolverine connectivity is an achievable goal. Furthermore, it is vital to recognize that with each additional parcel that is voluntarily transitioned into conservation, the mapping outcomes will change.

Conservation efforts are bolstered by including objectives that reflect the values of stakeholders and the affected communities (Gregory, 2000) and are a function of both stakeholder willingness and capacity to participate (Knight et al., 2010). Diverse motives drive private landholders to engage in conservation within dynamic social-ecological contexts (Gunningham and Young, 1997). However, existing gaps in communication and dissemination to stakeholders in many private land protection initiatives tend to hamper conservation success (Kamal et al., 2015; Capano et al., 2019). While this work provides important ecological information for conservation on private lands, local-scale engagement efforts will be necessary to ensure conservation actions are feasible for selected areas.

Private property rights are a sensitive issue of great financial, emotional, and cultural significance in the US. Two main approaches exist to balance wildlife conservation in the public domain with privately owned lands: 1) regulating private property and 2) incentivizing private property owners to achieve socially and beneficial ecological goals (Echeverria and Pidot, 2009). The first approach negatively affects rural landowners disproportionately (Ruhl, 1998) yield a positive rural attitude toward conserving wildlife. Thus, incentives that reward the landowner for maintaining rural lands in a more natural state that yields outcomes desirable by the larger society are essential for both rural community health and positive stakeholder conservation collaborations (Cheever and McLaughlin, 2004). This is particularly important in the West, where connectivity is critical for species persistence. The wolverine provides a clear example of the need to develop a network of privately owned natural areas that complement publicly owned lands.

### Complementary mitigating measures

Focusing on conservation actions with similar costs and probabilities of success is essential for robust prioritization (Game et al., 2013). This work focused solely on conservation easements on private lands because voluntary connectivity conservation is critical for mitigating anthropogenic effects on species globally. In the RMW, connecting wolverine habitat is likely to facilitate a network of connected wildlands that may benefit numerous other species and create a more resilient system of conservation areas (Belote et al., 2017). However, conservation easements are not the only viable solution to mitigate anthropogenic threats, and the associated incentives are generally more favorable to affluent landowners (Cheever and McLaughlin, 2004). For many species, wolverines included, other complementary mitigating measures can support and accelerate natural species recovery and reestablishment. In particular, complimentary mitigating measures that eliminate dispersal barriers would accelerate reestablishment and support species persistence in the current range. For example, there is evidence that large, multilane roads can be a barrier to wolverine dispersal and gene flow (Sawaya et al., 2019). Identifying potential sites for crossing structures within the area important for wolverine connectivity would bolster conservation efforts (Sijtsma et al., 2020). Alongside voluntary private land conservation, complementary mitigating measures, such as road crossings, will provide additional support for RMW species, but each mitigating measure should also undergo rigorous resource allocation prioritization.

### Limitations of study

We made several assumptions in our optimization model and output. One assumption was treating public lands and conservation easements as “fixed” and excluding them from our models. We excluded these areas because we considered public lands less susceptible to rapid land-use change and, therefore, somewhat protected. However, depending on ownership, each of these areas has varying degrees of protection and some level of human impact that could negatively influence wolverines, such as reclamation.

We also assumed that all landowners were equally likely to be interested in voluntary conservation easements. However, easements represent only one approach to engaging landowners in conservation efforts and are not equally appealing to all landowners. Further partnerships between agencies, land trusts, and social scientists are essential in implementing easements or alternative collaborative approaches for this work.

The final challenge inherent in landscape-scale optimization efforts is the presence of spatially dependent ecological thresholds, which considerably complicate prioritization analyses. Here, we treated the probability of use as continuous with cutoffs designed to reflect management needs rather than ecological thresholds for wolverine habitat use. However, in reality, a corridor will only be used if it consists of land with a sufficient amount and density of habitat (see Table S2). For this reason, we suggest our prioritization be used to aid conservation practitioners rather than an immutable authority in future implementation.

## STAR★Methods

### Key resources table

**Table:** 

REAGENT or RESOURCE	SOURCE	IDENTIFIER
**Software and algorithms**		

Circuitscape 4.0	McRae et al., 2008; McRae and Shah, 2009	https://circuitscape.org/
R Statistical Software	R Core Team 2020	https://www.r-project.org/
PriorizR	Hanson et al., 2019	https://prioritizr.net/
Gurobi	Gurobi Optimization, LLC	https://www.gurobi.com/
ArcMAP	ESRI	https://www.esri.com/

**Deposited data**		

Prioritization Shapefiles	This Paper	https://databasin.org/
Wolverine Telemetry Data	Inman et al., 2012	bobinman@mt.gov
Wolverine Occurrence Data	Lukacs et al., 2020	https://doi.org/10.1002/jwmg.21856
Wolverine Connectivity Data	Carroll et al., 2020	https://doi.org/10.1016/j.gecco.2020.e01125
Wolverine Core Habitat Data	Carroll et al., 2020	https://doi.org/10.1016/j.gecco.2020.e01125
Snow Water Equivalent	Integrated Scenarios Group	https://climate.northwestknowledge.net/IntegratedScenarios/
Human Modification	Theobald, 2013	https://databasin.org/datasets/110a8b7e238444e2ad95b7c17e889b66/
Cadastral Data	Montana Cadastral	http://svc.mt.gov/msl/mtcadastral

### Resource availability

#### Lead contact

Further information and requests should be directed to and will be fulfilled by the lead contact, Dr. Kathleen Carroll (kcarroll7@wisc.edu).

#### Materials availability

Shapefiles generated in this study have been deposited to Data Basin (https://databasin.org/).

#### Data and code availability

Restrictions apply to the availability of the wolverine data, which were used under contract for this study. Requests to access the dataset should be directed to Dr. Bob Inman, bobinman@mt.gov.

This paper does not report original code, see (https://prioritizr.net/).

Any additional information required to reanalyze the data reported in this paper is available from the lead contact upon request.

### Experimental model and subject details

Between 2001 and 2009, 38 wolverines (23 F, 15 M) from the GYE were fitted with intra-peritoneal VHF radio-transmitter collars. These animals were monitored for at least 3 years, and some individuals were monitored for up to 9 years (Inman et al., 2012). Eighteen (11 F, 7 M) of the original animals were also fitted with a global positioning system (GPS) collar for periods of ∼3 months, with relocations every 2 h. Location data were estimated to be accurate to within 300 m. The data collection was approved by the Animal Care and Use Committee of Montana Department of Fish, Wildlife and Parks.

### Method details

#### Study area

The Continental Divide bisects our study area in western Montana, and this area has a broad range of forest types and climatic regimes. Large portions of western Montana include state and federal lands administered by the National Park Service, U.S. Forest Service, Bureau of Land Management, US Fish and Wildlife Service, and Department of Defense. Exurban growth and development, shifting fire regimes, and climate change are among the greatest threats to connectivity and species across this region (Parks et al., 2005; Gude et al., 2006; Hansen and Phillips, 2018). Montana is approximately 63% private lands and 9% tribal lands and, like many other states in the RMW, has experienced a rapid transition from natural cover types to human-used landscapes. In some RMW ecosystems, over 50% of biophysical habitat types such as riparian and shrublands have been lost (Adhikari and Hansen, 2018). For example, the Greater Yellowstone Ecosystem experienced a 58% population increase and a 350% exurban housing increase between 1970 to 1999 (Gude et al., 2006), with exurban development continuing at a higher rate in many counties. Within the study area, 2083045.72 hectares of land was private land eligible for easements (Figure 1).

#### High-quality habitat

Our wolverine habitat models were based on previously collected wolverine relocation data (Inman et al., 2012) and 2040 to 2069 RCP 8.5 snow water equivalent (SWE) projections. We chose the SWE model because snow cover area, duration, and SWE are strong predictors of wolverine habitat (Schwartz et al., 2009; Carroll et al., 2020, but see Stewart et al., 2016). Additionally, the SWE data layer included a portion of the Rocky Mountains in southern Canada, which is ecologically important for wolverine population connectivity. There is gene flow between wolverines found in the US and Canada, and collared animals occasionally cross the border (Cegelski et al., 2006; COSEWIC 2014). We compared the SWE-only model to a previously analyzed model that included seven variables (Carroll et al., 2020). The SWE-only model predicted use and availability of withheld data with 78% accuracy (kappa = 32%). This score was 8 percentage points lower than the previously analyzed model. We determined that the importance of including habitat across the U.S.-Canada border and high SWE-only model performance justified using the SWE-only model for this analysis.

#### Connectivity analysis

We modeled wolverine habitat using a negative inverse relationship between future SWE habitat quality and resistance (Carroll et al., 2020) in Circuitscape 4.0 (McRae et al., 2008; McRae and Shah, 2009). We chose to use Circuitscape because it provides current density estimates representing both the paths individuals are likely to move and how tenuous movements between habitat cores are. In Circuitscape, connectivity values are highest along predicted routes or pathways when many random walkers pass through them. After generating our connectivity surface, we subset the surface to areas with the highest connectivity value (top 20%). We then prioritized lands within the high connectivity value area (connectivity value > 20%). Land outside of the high connectivity value area was not considered because preserving connectivity was the primary aim of the analysis. All prioritization analyses using additional variables were then conducted within this high connectivity study area.

### Quantification and statistical analysis

#### Characterizing variables and layers for prioritization

We included four variables to prioritize important land for wolverine conservation: current female presence, core size, human modification, and habitat core centrality (see Table S1). Genetic evidence of sex-biased dispersal and female philopatry in wolverines limits the recolonization of females’ previously occupied habitat (Tomasik and Cook, 2005). Given that female wolverines are less likely to disperse, areas that currently have female occupants are more immediately important for population persistence and reproduction (Lukacs et al., 2020). We also included core size in our prioritization framework to account for territoriality. In the western US, suitable and unsuitable conditions for wolverines exist in close proximity, and wolverines regularly patrol territorial boundaries, limiting the number of individuals in high-quality habitat (Inman et al., 2012). We considered patches that were larger extents of high-quality habitat to be more likely to support a larger number of individuals. We included human modification (Theobald, 2005, 2013) because human activities such as roads (Inman et al., 2013) and recreation (Krebs et al., 2007; Heinemeyer et al., 2019) impact wolverine habitat use. Finally, we considered centrality of linkages, or current flow centrality, a metric generated in the Linkage Mapper 2.0.0 Centrality Mapper (McRae and Kavanagh, 2011; McRae, 2012). Centrality identifies paths between cores that are critical to the maintenance of network connectivity. The centrality of linkage values allowed us to identify how vital any corridor between patches was in maintaining connectivity between areas of high-quality wolverine habitat.

#### Land ownership for Montana

We analyzed our prioritization model on western Montana private lands using parcel-level spatial data (Figure 1) from the US Public Land Survey System (www.cadastral.mt.gov). Areas were aggregated based on ownership (Figure 1). We designated conservation easements, state lands, and federal lands as “fixed” areas. These “fixed” areas were excluded from the optimization analysis as the scope of this work solely focused on areas where potential private landowner partners could engage in future conservation easement opportunities. We used 50% of the unique tax-assessed property value to assume the likely incentivized cost of a conservation easement and added a $5,000 transaction cost for each parcel (Ferraro, 2003).

#### Prioritization approach and tools

We prioritized areas using the prioritizR package in the program R (Hanson et al., 2019) with a Gurobi optimization solver. The prioritizR package uses integer linear programming (ILP) techniques to build conservation problems that can interface with several solver software algorithms and be spatially explicit (e.g., contiguity constraints can be included). Relatively similar approaches have been successfully applied to private lands for pronghorn (*Antilocapra americana*) and greater sage-grouse (*Centrocercus urophasianus*) migratory pathway conservation (Tack et al., 2019). Using this software, we analyzed four optimization targets, 0.10, 0.15, 0.20, and 0.50, representing the proportion of that variable optimized for each of the variables. Thus, in the 0.10 solution, land with the highest 10% of each optimization variable is identified. These targets allowed us to develop four output maps with the 10, 15, 20, and 50% of high-value areas in the study area while minimizing cost (i.e., rank of solution effectiveness is 10% > 15% > 20% > 50%). As the percentage of included high-value areas increases (e.g., from 10% to 15%), the added parcels are less immediately important for wolverine conservation. Thus, using four optimization targets enabled us to compare a hierarchy of important lands across Montana. After analyzing all four planning scenarios, we compared the land identified as important at the county level, as different easement holding land trusts may be interested in summary statistics specific to their jurisdiction.

## Supporting citations

Cardinal, 2004; Ceia-Hasse et al., 2017; Magoun, 1985; Persson et al., 2003; Powell, 1979; Rico et al., 2015; Theobald et al., 2012; US Geological Survey Gap Analysis Program, 20160513.

## References

[bib1] Adhikari A., Hansen A.J. (2018). Land use change and habitat fragmentation of wildland ecosystems of the North Central United States. Landscape Urban Plann..

[bib2] Ahmed S., Jackson-Smith D. (2019). Impacts of spatial patterns of rural and exurban residential development on agricultural trends in the intermountain west. SAGE Open.

[bib3] Allen C.H., Parrott L., Kyle C. (2016). An individual-based modelling approach to estimate landscape connectivity for bighorn sheep (*Ovis canadensis*). PeerJ.

[bib6] Aycrigg J.L., Davidson A., Svancara L.K., Gergely K.J., McKerrow A., Scott J.M. (2013). Representation of ecological systems within the protected areas network of the continental United States. PLoS One.

[bib7] Baier L.E. (2020). Saving Species on Private Lands: Unlocking Incentives to Conserve Wildlife and Their Habitats.

[bib8] Bargelt L., Fortin M.-J., Murray D.L. (2020). Assessing connectivity and the contribution of private lands to protected area networks in the United States. PLoS One.

[bib9] Belote R.T., Dietz M.S., Jenkins C.N., McKinley P.S., Irwin G.H., Fullman T.J., Leppi J.C., Aplet G.H. (2017). Wild, connected, and diverse: building a more resilient system of protected areas. Ecol. Appl..

[bib10] Beyer H.L., Dujardin Y., Watts M.E., Possingham H.P. (2016). Solving conservation planning problems with integer linear programming. Ecol. Model..

[bib11] Boyd J., Epanchin-Niell R., Siikamäki J. (2015). Conservation planning: a review of return on investment analysis. Rev. Environ. Econ. Pol..

[bib12] Braunstein J.L., Clark J.D., Williamson R.H., Stiver W.H. (2020). Black bear movement and food conditioning in an exurban landscape. J. Wildl. Manage..

[bib14] Brown C.J., Bode M., Venter O., Barnes M.D., McGowan J., Runge C.A., Watson J.E., Possingham H.P. (2015). Effective conservation requires clear objectives and prioritizing actions, not places or species. Proc. Natl. Acad. Sci. U S A.

[bib15] Capano G.C., Toivonen T., Soutullo A., Di Minin E. (2019). The emergence of private land conservation in scientific literature: a review. Biol. Conserv..

[bib16] Cardinal N. (2004). Aboriginal Traditional Knowledge COSEWIC Status Report on Wolverine *Gulo gulo Qavvik*.

[bib17] Carroll K.A., Hansen A.J., Inman R.M., Lawrence R.L., Hoegh A.B. (2020). Testing landscape resistance layers and modeling connectivity for wolverines in the western United States. Glob. Ecol. Conserv..

[bib18] Carter S.K., Januchowski-Hartley S.R., Pohlman J.D., Bergeson T.L., Pidgeon A.M., Radeloff V.C. (2015). An evaluation of environmental, institutional and socioeconomic factors explaining successful conservation plan implementation in the north-central United States. Biol. Conserv..

[bib21] Cegelski C., Waits L., Anderson N., Flagstad O., Strobeck C., Kyle C. (2006). Genetic diversity and population structure of wolverine (*Gulo gulo*) populations at the southern edge of their current distribution in North America with implications for genetic viability. Conserv. Genet..

[bib22] Ceia-Hasse A., Borda-de-Água L., Grilo C., Pereira H.M. (2017). Global exposure of carnivores to roads. Glob. Ecol. Biogeogr..

[bib23] Cheever F., McLaughlin N.A. (2004). Why environmental lawyers should know (and care) about land trusts and their private land conservation transactions. Envtl. L. Rep. News & Analysis.

[bib24] [COSEWIC] Committee on the Status of Endangered Wildlife in Canada (2014). COSEWIC Assessment and Status Report on the Wolverine (*Gulo gulo*) in Canada. Ottowa, Canada. https://www.sararegistry.gc.ca/virtual_sara/files/cosewic/sr_Wolverine_2014_e.pdf.

[bib26] Dietz R.W., Czech B. (2005). Conservation deficits for the continental United States: an ecosystem gap analysis. Conserv. Biol..

[bib27] Dilkina B., Houtman R., Gomes C.P., Montgomery C.A., McKelvey K.S., Kendall K., Graves T.A., Bernstein R., Schwartz M.K. (2017). Trade-offs and efficiencies in optimal budget-constrained multispecies corridor networks. Conserv. Biol..

[bib28] Echeverria J., Pidot J. (2009). Drawing the line: striking a principled balance between regulating and paying to protect the land. Environ. L. Reporter News Anal..

[bib29] Elbroch L.M., Ferguson J.M., Quigley H., Craighead D., Thompson D.J., Wittmer H.U. (2020). Reintroduced wolves and hunting limit the abundance of a subordinate apex predator in a multi-use landscape. Proc. R. Soc. B.

[bib31] Ferraro P.J. (2003). Assigning priority to environmental policy interventions in a heterogeneous world. J. Policy Anal. Manage..

[bib32] Game E.T., Kareiva P., Possingham H.P. (2013). Six common mistakes in conservation priority setting. Conserv. Biol..

[bib34] Gregory R. (2000). Using stakeholder values to make smarter environmental decisions. Environ. Sci. Pol. Sustain. Dev..

[bib35] Gude P.H., Hansen A.J., Rasker R., Maxwell B. (2006). Rates and drivers of rural residential development in the Greater Yellowstone. Landscape Urban Plann..

[bib36] Gunningham N., Young M.D. (1997). Toward optimal environmental policy: the case of biodiversity conservation. Ecol. Law Q..

[bib37] Haenel G.J., Smith L.C., John-Alder H.B. (2003). Home-range analysis in *Sceloporus undulatus* (eastern fence lizard). I. Spacing patterns and the context of territorial behavior. Copeia.

[bib38] Hamilton C.M., Baumann M., Pidgeon A.M., Helmers D.P., Thogmartin W.E., Heglund P.J., Radeloff V.C. (2016). Past and predicted future effects of housing growth on open space conservation opportunity areas and habitat connectivity around National Wildlife Refuges. Landscape Ecol..

[bib39] Hansen A.J., Phillips L. (2018). Trends in vital signs for greater Yellowstone: application of a wildland health Index. Ecosphere.

[bib40] Hanson J., Schuster R., Morrell N., Strimas-Mackey M., Watts M., Arcese P., Bennett J., Possingham H. (2019). Prioritizr: Systematic Conservation Prioritization in R. R Package Version 4.1.4.

[bib41] Heinemeyer K., Squires J., Hebblewhite M., O’Keefe J.J., Holbrook J.D., Copeland J. (2019). Wolverines in winter: indirect habitat loss and functional responses to backcountry recreation. Ecosphere.

[bib89] Inman R.M., Brock B.L., Inman K.H., Sartorius S.S., Aber B.C., Giddings B., Cain S.L., Orme M.L., Fredrick J.A., Oakleaf B.J., Alt K.L. (2013). Developing priorities for metapopulation conservation at the landscape scale: wolverines in the western United States. Biological Conservation.

[bib44] Inman R.M., Packila M.L., Inman K.H., Mccue A.J., White G.C., Persson J., Aber B.C., Orme M.L., Alt K.L., Cain S.L. (2012). Spatial ecology of wolverines at the southern periphery of distribution. J. Wildl. Management.

[bib45] Johnson H.E., Sushinsky J.R., Holland A., Bergman E.J., Balzer T., Garner J., Reed S.E. (2017). Increases in residential and energy development are associated with reductions in recruitment for a large ungulate. Glob. Change Biol..

[bib46] Joppa L.N., Pfaff A. (2009). High and far: biases in the location of protected areas. PLoS One.

[bib47] Kamal S., Kocór M., Grodzińska-Jurczak M. (2015). Conservation opportunity in biodiversity conservation on regulated private lands: factors influencing landowners’ attitude. Environ. Sci. Pol..

[bib48] Knight A.T., Cowling R.M. (2007). Embracing opportunism in the selection of priority conservation areas. Conserv. Biol..

[bib49] Knight A.T., Cowling R.M., Difford M., Campbell B.M. (2010). Mapping human and social dimensions of conservation opportunity for the scheduling of conservation action on private land. Conserv. Biol..

[bib50] Krebs J., Lofroth E.C., Parfitt I. (2007). Multiscale habitat use by wolverines in British Columbia, Canada. J. Wildl. Management.

[bib51] Kyle C.J., Strobeck C. (2001). Genetic structure of North American wolverine (*Gulo gulo*) populations. Mol. Ecol..

[bib52] Lukacs P.M., Evans Mack D., Inman R., Gude J.A., Ivan J.S., Lanka R.P., Lewis J.C., Long R.A., Sallabanks R., Walker Z. (2020). Wolverine occupancy, spatial distribution, and monitoring design. J. Wildl. Management.

[bib53] Magoun A.J. (1985). Population Characteristics, Ecology, and Management of Wolverines in Northwestern Alaska (*Gulo-Gulo*).

[bib54] Margules C.R., Pressey R.L. (2000). Systematic conservation planning. Nature.

[bib55] McRae B. (2012). Centrality Mapper Connectivity Analysis Software.

[bib56] McRae B.H., Dickson B.G., Keitt T.H., Shah V.B. (2008). Using circuit theory to model connectivity in ecology, evolution, and conservation. Ecology.

[bib57] McRae B.H., Kavanagh D.M. (2011). Linkage mapper connectivity analysis software. https://code.google.com/p/linkage-mapper/.

[bib58] McRae B.H., Shah V.B. (2009). Circuitscape User’s Guide.

[bib59] Middleton A.D., Sawyer H., Merkle J.A., Kauffman M.J., Cole E.K., Dewey S.R., Gude J.A., Gustine D.D., McWhirter D.E., Proffitt K.M. (2020). Conserving transboundary wildlife migrations: recent insights from the Greater Yellowstone Ecosystem. Front. Ecol. Environ..

[bib60] Naidoo R., Balmford A., Ferraro P.J., Polasky S., Ricketts T.H., Rouget M. (2006). Integrating economic costs into conservation planning. Trends Ecol. Evol..

[bib62] Parks C.G., Radosevich S.R., Endress B.A., Naylor B.J., Anzinger D., Rew L.J., Maxwell B.D., Dwire K.A. (2005). Natural and land-use history of the Northwest mountain ecoregions (USA) in relation to patterns of plant invasions. Perspect. Plant Ecol. Evol. Syst..

[bib64] Perry G.L., Lee F. (2019). How does temporal variation in habitat connectivity influence metapopulation dynamics?. Oikos.

[bib65] Persson J., Willebrand T., Landa A., Andersen R., Segerström P. (2003). The role of intraspecific predation in the survival of juvenile wolverines Gulo gulo. Wildl. Biol..

[bib67] Powell R.A. (1979). Mustelid spacing patterns: variations on a theme by Mustela. Z. für Tierpsychol..

[bib68] Quintas-Soriano C., Gibson D.M., Brandt J.S., López-Rodríguez M.D., Cabello J., Aguilera P.A., Castro A.J. (2020). An interdisciplinary assessment of private conservation areas in the Western United States. Ambio.

[bib90] R Core Team (2020). R: A language and environment for statistical computing. https://www.R-project.org/.

[bib69] Rico Y., Morris-Pocock J., Zigouris J., Nocera J.J., Kyle C.J. (2015). Lack of spatial immunogenetic structure among wolverine (Gulo gulo) populations suggestive of broad scale balancing selection. PLoS One.

[bib70] Ruhl J.B. (1998). Endangered species act and private property: a matter of timing and location. Cornell J. Law Public Policy.

[bib71] Runge C.A., Plantinga A.J., Larsen A.E., Naugle D.E., Helmstedt K.J., Polasky S., Donnelly J.P., Smith J.T., Lark T.J., Lawler J.J. (2019). Unintended habitat loss on private land from grazing restrictions on public rangelands. J. Appl. Ecol..

[bib72] Sacre E., Pressey R.L., Bode M. (2019). Costs are not necessarily correlated with threats in conservation landscapes. Conserv. Lett..

[bib73] Sawaya M.A., Clevenger A.P., Schwartz M.K. (2019). Demographic fragmentation of a protected wolverine population bisected by a major transportation corridor. Biol. Conserv..

[bib74] Sawyer H., Kauffman M.J., Nielson R.M., Horne J.S. (2009). Identifying and prioritizing ungulate migration routes for landscape-level conservation. Ecol. Appl..

[bib75] Schwartz M.K., Copeland J.P., Anderson N.J., Squires J.R., Inman R.M., McKelvey K.S., Pilgrim K.L., Waits L.P., Cushman S.A. (2009). Wolverine gene flow across a narrow climatic niche. Ecology.

[bib76] Scott J.M., Davis F.W., McGhie R.G., Wright R.G., Groves C., Estes J. (2001). Nature reserves: do they capture the full range of America’s biological diversity?. Ecol. Appl..

[bib78] Sijtsma F.J., van der Veen E., van Hinsberg A., Pouwels R., Bekker R., van Dijk R.E., Grutters M., Klaassen R., Krijn M., Mouissie M. (2020). Ecological impact and cost-effectiveness of wildlife crossings in a highly fragmented landscape: a multi-method approach. Landscape Ecol..

[bib79] Smith J., Evans J., Martin B., Baruch-Mordo S., Kiesecker J., Naugle D. (2016). Reducing cultivation risk for at-risk species: Predicting outcomes of conservation easements for sage-grouse. Biol. Conserv..

[bib80] Stewart F.E., Heim N.A., Clevenger A.P., Paczkowski J., Volpe J.P., Fisher J.T. (2016). Wolverine behavior varies spatially with anthropogenic footprint: implications for conservation and inferences about declines. Ecol. Evol..

[bib81] Tack J.D., Jakes A.F., Jones P.F., Smith J.T., Newton R.E., Martin B.H., Hebblewhite M., Naugle D.E. (2019). Beyond protected areas: private lands and public policy anchor intact pathways for multispecies wildlife migration. Biol. Conserv..

[bib82] Theobald D.M. (2005). Landscape patterns of exurban growth in the USA from 1980 to 2020. Ecol. Soc..

[bib83] Theobald D.M. (2013). A general model to quantify ecological integrity for landscape assessments and US application. Landscape Ecol..

[bib84] Theobald D.M., Reed S.E., Fields K., Soulé M. (2012). Connecting natural landscapes using a landscape permeability model to prioritize conservation activities in the United States. Conserv. Lett..

[bib85] Tomasik E., Cook J.A. (2005). Mitochondrial phylogeography and conservation genetics of wolverine (*Gulo gulo*) of northwestern North America. J. Mammal..

[bib86] US Geological Survey Gap Analysis Program (2016). GAP/LANDFIRE national terrestrial ecosystems 2011. US Geol. Surv..

