# Supplementary material for: A framework for collaborative wolverine connectivity conservation
Source: iScience. 2021 Jul 10;24(8):102840. doi: 10.1016/j.isci.2021.102840 (PMC8326200; doi:10.1016/j.isci.2021.102840)
Supplement: Document S1. Tables S1 and S2 [file mmc1.pdf]

iScience, Volume *24*

Supplemental information

A framework for collaborative wolverine connectivity conservation

Kathleen A. Carroll, Robert M. Inman, Andrew J. Hansen, Rick L. Lawrence, and Kevin Barnett

Supplemental Tables and Figures

Table S1. Related to STAR Methods. The evidence supporting the inclusion of each variable in the prioritization framework.

Variable	Relevance	Data Source	Evidence	Proposed Framework	Strength of the Evidence for Wolverine Conservation
Core Size	– larger areas of high-quality habitat support a larger number of individuals given wolverine intrasexual territoriality	This analysis; Lukacs et al., 2020	(Powell, 1979; Magoun, 1985; Persson et al., 2003; Inman et al., 2012)	Prioritize the protection of larger extents of high-quality wolverine habitat likely to support a larger number of individuals.	Strong – the literature has consistently demonstrated the importance of high-quality breeding habitat.
Female Presence Data	– limited recolonization of previously occupied habitat based on genetic evidence of sex-biased dispersal and female philopatry in wolverines<br>– areas that currently have female occupants are more valuable to ensure population persistence and reproduction	This analysis; (Lukacs et al., 2020)	(Rico et al., 2015)	Prioritize areas where female wolverines are likely to be reproducing and supporting population growth.	Moderate – the literature indicates the importance of connecting habitat for sex-biased dispersal. However, the non-invasive genetic samples data is based on only 240 detections across 4 states.

Current Flow Centrality (CFC)	– identifies how vital any corridor between patches was in maintaining connectivity<br>– provides an analytical method for prioritizing connectivity	This analysis	(McRae et al., 2008; Theobald et al., 2012; Osipova et al., 2018)	Prioritize areas that are more important in preserving linkages between cores.	Moderate – literature demonstrates that CFC is useful for prioritizing connectivity, but this approach relies on LCP network, which does not predict wolverine dispersal as well as circuit theory (McClure et al., 2016).
Road Density	– carnivores (including Mustelidae) have higher contact with roads than other mammals<br>– roads are a barrier that contributes to genetic isolation in wolverines	2018 TIGER/Line Data (US Census Bureau)	(Ceia-Hasse et al., 2017; Sawaya et al., 2019)	Prioritize areas with low road densities.	Weak – limited evidence that wolverines are limited by road density.
Human Modification	– Wolverine movement within-home range is impacted by human activities; such as recreation<br>– Aboriginal knowledge holders in Canada report	(Theobald 2005; Theobald, 2013)	(Cardinal, 2004; Krebs et al., 2007; Heinemeyer et al., 2019)	prioritize rural landscapes	Moderate –it has proven difficult to test whether wolverines avoid human infrastructure; due to the general lack of infrastructure within otherwise suitable

	wolverines avoid areas with human development		wolverine habitat. However; it is reasonable to assume that wolverine dispersal habitat quality lowers with increasing infrastructure

Table S2. Related to Figure 2. We also examined the proportion of land cover types in solution (US Geological SurveyGap Analysis Program, 2016). Given the broad study area and the adverse effects of fragmentation on ecological processes, it is important to understand what land-use types are prioritized. Additionally, threats and conservation action should vary by land tenure and associated land protections. In addition to the land tenure, we examined broad land cover types for easements. Cool semi-desert scrub and grassland, temperate grassland and shrubland, and cool temperate forest and woodland were the most abundant land cover types for all solutions. Other land cover types, including recently disturbed areas, hay crop fields, wet meadows and shrubland, and tundra, were identified. GAP land cover classification of all parcels identified in each solution. Land cover types with fewer than 100 ha were excluded from the table.

Priority Level	Land Cover	Area (ha)	Aggregate Percent of Total Area
10%	Cool Semi-Desert Scrub & Grassland	45371.74	0.40
	Temperate Grassland & Shrubland	37676.37	0.73
	Cool Temperate Forest & Woodland	16853.64	0.88
	Pasture & Hay Field Crop	4183.99	0.92
	Temperate to Polar Freshwater Marsh; Wet Meadow & Shrubland	2302.62	0.94

	Recently Disturbed or Modified	2287.65	0.96
	Temperate & Boreal Alpine Tundra	1814.73	0.98
	Developed & Urban	1161.10	0.99
15%	Cool Semi-Desert Scrub & Grassland	65816.30	0.38
	Temperate Grassland & Shrubland	55751.70	0.71
	Cool Temperate Forest & Woodland	28809.73	0.88
	Pasture & Hay Field Crop	6972.14	0.92
	Temperate to Polar Freshwater Marsh; Wet Meadow & Shrubland	4050.09	0.94
	Recently Disturbed or Modified	3543.94	0.96
	Developed & Urban	1832.10	0.98
	Temperate & Boreal Alpine Tundra	1815.96	0.99
	Row & Close Grain Crop Cultural Formation	1338.24	0.99
	Temperate Flooded & Swamp Forest	1066.62	1.00
20%	Cool Semi-Desert Scrub & Grassland	83869.02	0.36
	Temperate Grassland & Shrubland	73740.71	0.68
	Cool Temperate Forest & Woodland	43662.36	0.87
	Pasture & Hay Field Crop	10756.91	0.91
	Recently Disturbed or Modified	5899.31	0.94
	Temperate to Polar Freshwater Marsh; Wet Meadow & Shrubland	5778.97	0.96
	Developed & Urban	2512.89	0.98
	Temperate & Boreal Alpine Tundra	2179.92	0.98
	Row & Close Grain Crop Cultural Formation	2031.63	0.99
	Temperate Flooded & Swamp Forest	1300.42	1.00
50%	Cool Semi-Desert Scrub & Grassland	181561.78	0.30
	Temperate Grassland & Shrubland	178097.91	0.59
	Cool Temperate Forest & Woodland	150535.51	0.84
	Pasture & Hay Field Crop	40359.51	0.90
	Temperate to Polar Freshwater Marsh; Wet Meadow & Shrubland	19637.61	0.93
	Recently Disturbed or Modified	17061.97	0.96
	Row & Close Grain Crop Cultural Formation	9125.85	0.98
	Developed & Urban	6746.69	0.99
	Temperate Flooded & Swamp Forest	4785.17	1.00
	Temperate & Boreal Alpine Tundra	2347.40	1.00
